# Preoperative Contrast-enhanced CT Features Associated with Occult Lymph Node Metastasis in Early-Stage Solid Non–Small Cell Lung Cancer

**DOI:** 10.1148/rycan.250448

**Published:** 2026-03-06

**Authors:** Yuyi Feng, Huangqi Zhang, Jiaqian Yu, Lingxia Wang, Yitian Wu, Lingwei Zhu, Jianchen Zheng, Ying Chen, Jincheng Lai, Hai Yang, Tao-Hsin Tung, Minghui Cai, Wenbin Ji

**Affiliations:** ^1^School of Medicine, Shaoxing University, Shaoxing 312000, China; ^2^Department of Radiology, Taizhou Hospital of Zhejiang Province affiliated to Wenzhou Medical University, Linhai 317000, Zhejiang, China; ^3^Department of Radiology, Taizhou Hospital of Zhejiang Province, Zhejiang University, Taizhou, China; ^4^College of Basic Medicine and Forensic Medicine, Hangzhou Medical College, Hangzhou, China; ^5^Evidence-based Medicine Center, Taizhou Hospital of Zhejiang Province Affiliated to Wenzhou Medical University, Taizhou, China; ^6^Department of Cardiothoracic Surgery, Taizhou Hospital of Zhejiang Province affiliated to Wenzhou Medical University, Linhai, China; ^7^Key Laboratory of Evidence-based Radiology of Taizhou, Linhai, China

**Keywords:** Imaging Modality, Lung, Neoplasms-Primary, Thorax

## Abstract

**Purpose:**

To develop and validate a contrast-enhanced CT-based prediction model for identifying occult lymph node metastasis (OLNM) in patients with early-stage non–small cell lung cancer (NSCLC), with the goal of supporting individualized lymph node dissection (LND) strategies.

**Materials and Methods:**

This retrospective study included patients with preoperative clinical stage I–IIA (cT1–T2bN0M0) solid NSCLC who underwent lobectomy with systematic LND between January 2021 and April 2024. Univariable and multivariable logistic regression analyses were used to identify independent preoperative CT predictors of OLNM and to construct a nomogram. Model performance was assessed using the area under the receiver operating characteristic curve, and specificity was evaluated at a fixed sensitivity of 95%.

**Results:**

Among 329 patients with solid NSCLC (median age, 65 years; IQR, 58–70 years; 168 male patients), 22.2% (73 of 329) had OLNM, including 47.9% (35 of 73) with N1 and 52.1% (38 of 73) with N2 metastases. Independent predictors of OLNM were a decreased inner margin ratio (odds ratio [OR], 0.02; 95% CI: 0.00, 0.10; *P* < .001), presence of the lollipop sign (OR, 3.48; 95% CI: 1.87, 6.49; *P* < .001), and tumor-pleura relationship type II (OR, 6.95; 95% CI: 2.62, 18.44; *P* < .001) and type III (OR, 13.27; 95% CI: 5.11, 34.45; *P* < .001). The nomogram achieved an area under the receiver operating characteristic curve of 0.81 (95% CI: 0.76, 0.87), with a sensitivity of 78.1% and specificity of 73.4%; specificity was 39.1% at 95% sensitivity.

**Conclusion:**

A contrast-enhanced CT-based nomogram incorporating inner margin ratio, lollipop sign, and tumor-pleura relationship enabled effective preoperative risk stratification for OLNM in early-stage solid NSCLC and may aid in tailoring LND strategies.

**Keywords:** Imaging Modality, Lung, Neoplasms-Primary, Thorax

*Supplemental material is available for this article.*

© The Author(s) 2026. Published by the Radiological Society of North America under a CC BY 4.0 license.

## Graphical abstract

**Figure:**
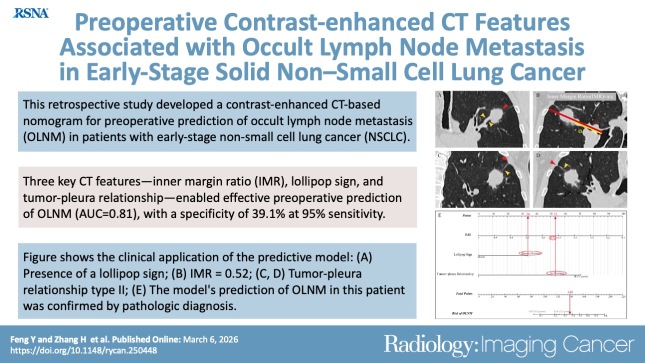

**Summary**

Inner margin ratio, lollipop sign, and tumor-pleura relationship on preoperative contrast-enhanced CT scans independently predicted occult lymph node metastasis in patients with early-stage solid non–small cell lung cancer.

**Key Points**

■ In a retrospective study of 329 patients with early-stage non–small cell lung cancer, occult lymph node metastasis (OLNM) was identified in 73 patients (22.2%) using postoperative pathology.
■ Lower inner margin ratio (odds ratio [OR], 0.02), presence of a lollipop sign (OR, 3.48), and tumor-pleura relationship types II (OR, 6.95) and III (OR, 13.27) at preoperative contrast-enhanced CT independently predicted OLNM (all *P* < .001).
■ A nomogram incorporating these CT scan features achieved good performance for predicting OLNM (area under the receiver operating characteristic curve, 0.81), with a specificity of 39.1% at 95% sensitivity.

## Introduction

The most common cancer diagnosed worldwide is lung cancer (1), for which the highest incidence rate and number of deaths occur in China (2). Approximately 85% of all lung tumors are non–small cell lung cancers (NSCLC) (3). Since Cahan (4) proposed the definition of lobectomy (surgical excision of lung lobe) in 1960, lobectomy with systematic lymph node dissection (LND) has become the standard surgical procedure for the treatment of early-stage lung cancer (5). Although the criterion for classifying a negative lymph node is a short-axis diameter of 10 mm or less on preoperative CT images (6), approximately 11.2%–18.5% of patients classified as N0 at preoperative imaging were found to have pathologic lymph node metastases (7–9), known as occult lymph node metastasis (OLNM).

Lymph node involvement is closely associated with poor prognosis in NSCLC; therefore, the current guidelines, such as those of the National Comprehensive Cancer Network and the European Society for Medical Oncology (10–11), recommend systematic rather than selective LND in patients with early-stage lung cancer. The current LND strategies, however, are controversial (12), with the Z0030 trial by the American College of Surgeons Oncology Group (13) finding that systematic and selective LNDs resulted in similar survival rates. Although selective LND is becoming more widespread, an alternative treatment—stereotactic body radiation therapy—has emerged for patients who are not surgical candidates (14). Some metastatic lymph nodes, however, may be overlooked (15), which is a potential risk factor for postoperative recurrence. This could lead to a poor prognosis (16), as excessive LND increases the risk of postoperative complications (17,18). Nonmetastatic lymph nodes may play an important role in antitumor immunity and modulate antitumor responses to immunotherapy (19); therefore, the accurate preoperative identification of patients with OLNM is crucial to determine the extent of LND.

The American College of Chest Physicians evidence-based clinical practice guidelines illustrate that the specificity and sensitivity of CT scan features in the assessment of lymph node status are 81% and 55%, respectively (20). As reported in previous studies, lymph node imaging features are not predictive of OLNM (21); therefore, it is necessary to focus on primary tumor, not lymph node, features to predict the risk of OLNM in patients with early-stage NSCLC. Previous studies have found that the following noncontrast-enhanced CT scan features are related to OLNM: tumor size (22), lobulation, spiculation (7), tumor-pleura relationship (abutting on the pleura with a broad base) (21), a consolidation to tumor ratio of more than 0.75 (8), and inner margin ratio (IMR) (23). However, not only does contrast-enhanced CT show additional features, such as blood supply and the enhancement pattern of the primary tumor, but it also has the advantage of accurately identifying lymph nodes that are not well-visualized at noncontrast-enhanced CT.

This study aimed to develop a preoperative risk model for OLNM in patients with early-stage NSCLC using contrast-enhanced CT imaging features to support risk stratification and treatment decision-making. To this end, three key CT scan features were investigated: *(a)* IMR (23), defined as the ratio of the distance from the lobar bronchus to the inner tumor margin relative to the total distance from the lobar bronchus to the pleura; *(b)* lollipop sign (24), characterized by a single, uniformly thickened blood vessel penetrating into the center of the nodule; and *(c)* tumor-pleura relationship (25), classified as type I (no pleural contact or linear pleural tags without pleural indentation), type II (linear pleural tags with pleural indentation), or type III (broad-based adhesion to the pleura).

## Materials and Methods

This study was approved by the institutional review board at Taizhou Hospital of Zhejiang Province (no. K20250411), and the requirement for informed consent was waived, owing to the retrospective nature of the study.

### Study Patients

This retrospective observational cohort study analyzed data at the patient level.

**Figure 1:**
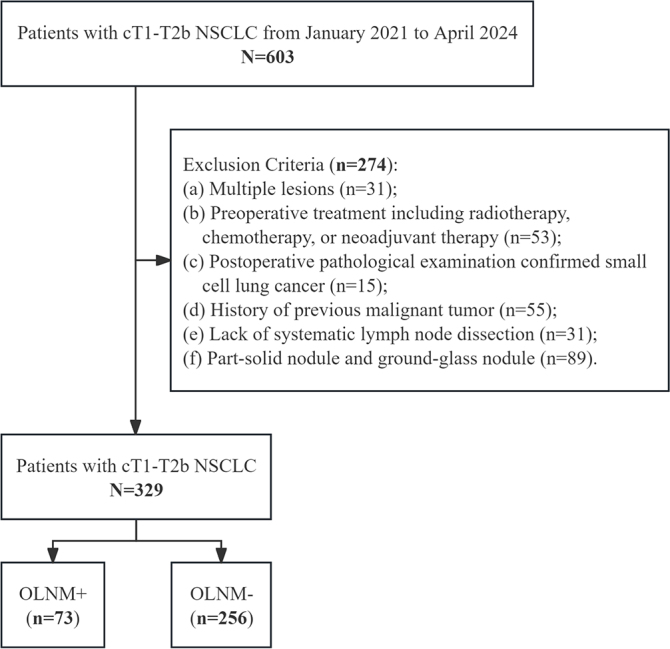
Flow diagram of study patients. NSCLC = non–small cell lung cancer. OLNM = occult lymph node metastasis.

Consecutive patients with preoperative clinical stage I–IIA (cT1–T2bN0M0) NSCLC confirmed through surgical resection at Taizhou Hospital of Zhejiang Province between January 2021 and April 2024 (International Association for the Study of Lung Cancer, ninth edition) were retrospectively included in this study. The inclusion criteria were as follows: *(a)* patients underwent lobectomy and systematic LND; *(b)* contrast-enhanced CT of the chest obtained less than 1 month before surgery; and *(c)* a lymph node short diameter of less than 1 cm on preoperative contrast-enhanced CT images. Exclusion criteria included multiple lung lesions, preoperative treatment (radiation therapy, chemotherapy, or neoadjuvant therapy), pathologically confirmed small cell lung cancer, previous malignant tumor, lack of systematic LND, and part-solid nodules or ground-glass nodules. Detailed inclusion and exclusion criteria are shown in Figure 1. All variables included in the final analysis were complete, with no missing data. N1 and N2 nodal status were determined according to the TNM classification (ninth edition). Systematic LND was defined as the removal of all lymphatic tissue from each of the multiple lymph node stations, including at least three mediastinal stations (N2) and a minimum of six lymph nodes in total (26). OLNM-positive status was defined as positive lymph node metastasis confirmed postoperatively by pathologic evaluation of the resected specimen, with a maximum short diameter of less than 1 cm observed at preoperative contrast-enhanced CT.

### Contrast-enhanced Chest CT

A 64-slice CT scanner (Discovery CT750 HD; GE HealthCare) was used to evaluate patients who underwent routine respiration training. The scanning parameters were as follows: tube voltage, 120 kV; tube current (Smart mA), 100–200 mA; rotation time, 0.6 second; field of view, 300–400 mm; pixel matrix size, 512 × 512; section thickness and interval, 5.0 mm; contrast agent injection rate, 2.5–3.0 mL/sec; and injection volume, 1.1 mL/kg. Arterial and venous phase sequences were initiated 25 and 60 seconds after the contrast agent injection. A thin-layer postprocessing reconstruction was performed for the axial, sagittal, and coronal planes after each scan.

### CT Scan Features for Predicting OLNM

All CT studies were independently evaluated for enhanced and unenhanced tumor imaging features by two radiologists (J.Y. and Y.F., with 10 and 4 years of experience, respectively, in diagnostic chest imaging) who were blinded to the relevant clinicopathologic data. Any disagreements were adjudicated by a third senior radiologist (H.Y., with over 20 years of experience in diagnostic chest imaging), and final decisions were reached by consensus. Solid nodules were characterized by a solid component comprising 80% or more of the nodule’s diameter, according to the International Early Lung Cancer Action Program Investigator Group (27).

### IMR, Outer Margin Ratio, and Inner Margin Location

**Figure 2:**
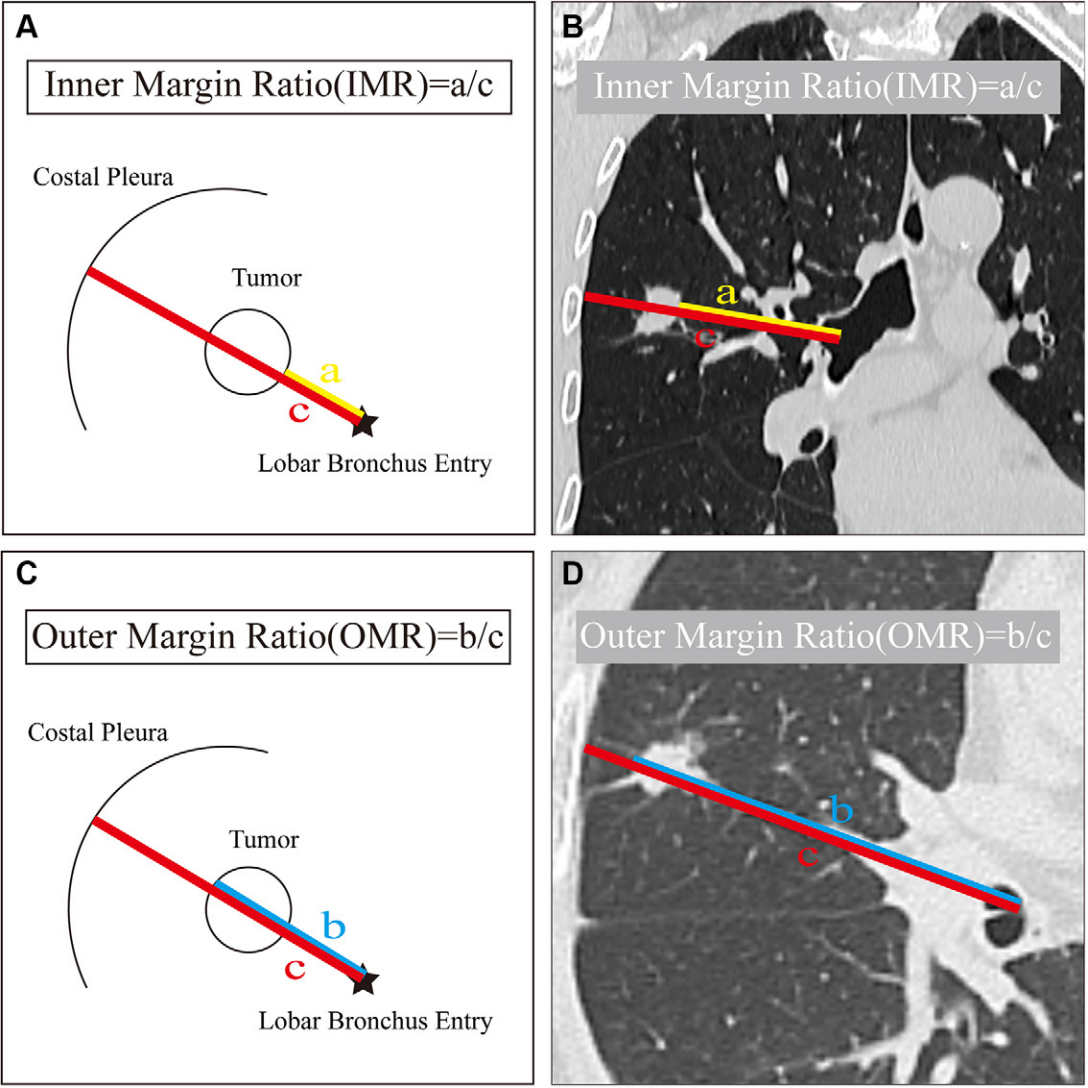
Detailed measurements of inner margin ratio (IMR) and outer margin ratio (OMR) on three-dimensional multiplanar reconstruction images. IMR was calculated as the ratio of distance a (from the lobar bronchus entry point to the inner tumor margin) to distance c (from the bronchus entry point to the pleura). Similarly, OMR was calculated as the ratio of distance b (to the outer tumor margin) to distance c. IMR and OMR are shown in **(A, C)** pictograms and **(B, D)** coronal and axial CT images. **(B)** Coronal image in a 56-year-old male patient with an IMR of 0.63 (66.6 mm/105.7 mm) in the right upper lobe. **(D)** Axial image in a 65-year-old male patient with an OMR of 0.87 (88.5 mm/102.2 mm) in the right middle lobe. All measurements were performed twice by a single observer with a 2-week interval, and the average value was used for analysis.

To describe the location of the tumor within the lung lobe on three-dimensional multiplanar reconstruction images, IMR and outer margin ratio (OMR) were calculated as follows (see Fig 2): IMR is the distance from the lobar bronchus to the tumor’s inner margin divided by the distance from the lobar bronchus to the pleura. OMR is the distance from the lobar bronchus to the tumor’s outer margin divided by the distance from the lobar bronchus to the pleura. The inner margin location for each tumor was categorized based on IMR as medial (IMR, 0–0.33), intermedius (IMR, 0.34–0.67), or lateral (IMR, 0.68–1).

### Tumor-Pleura Relationship

**Figure 3:**
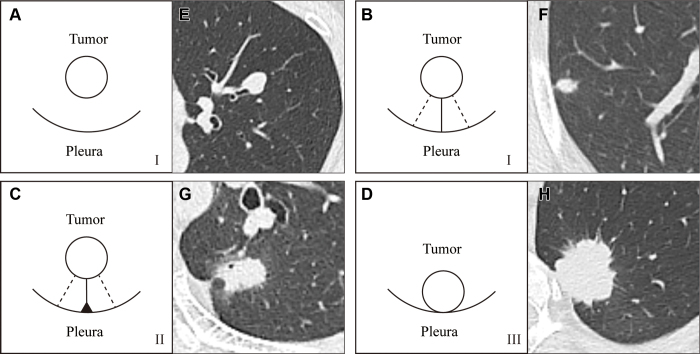
Schematic diagram of tumor-pleura relationship. Tumor-pleura relationship is shown in **(A–D)** pictograms and **(E–H)** axial CT images. **(A)** and **(B)** correspond to type I, **(C)** corresponds to type II, and **(D)** corresponds to type III. **(E)** Axial image in a 61-year-old female patient’s nodule with no contact with pleura. **(F)** Axial image in a 58-year-old male patient’s nodule with one linear pleural tag. **(G)** Axial image in a 61-year-old male patient’s nodule with one linear pleural tag and pleural indentation. **(H)** Axial image in a 66-year-old female patient’s nodule with a wide base adhered to the pleura.

Tumor-pleura relationships (Fig 3) were classified into the following three categories: I, the tumor had no contact with pleura or had one or more linear pleural tags with no pleural indentation; II, the tumor had one or more linear pleural tags with pleural indentation; and III, the tumor adhered to the pleura with a wide base (25).

### Lollipop Sign

**Figure 4:**
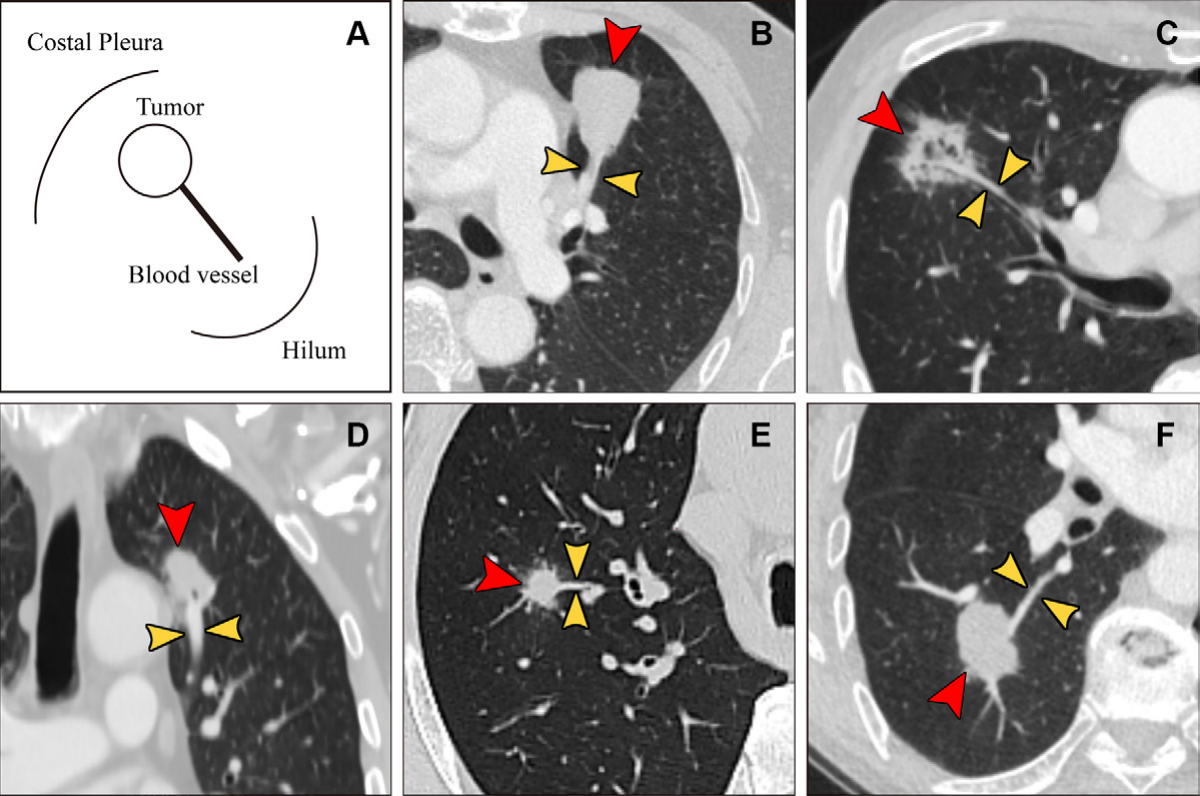
Representative images of a lollipop sign. A lollipop sign, defined as a single, uniformly thickened blood vessel emanating from the pulmonary hilum and penetrating into the middle of the lung nodule on any multiplanar reconstructive CT arterial phase image, is shown in **(A)** pictograms and **(B–F)** coronal and axial CT images. On the CT images, the red arrowheads indicate the lung nodule, and the yellow arrowheads indicate the single blood vessel entry. **(B)** Axial image in a 72-year-old female patient with adenocarcinoma in the left upper lobe. **(C)** Axial image in a 70-year-old male patient with squamous cell carcinoma in the right upper lobe. **(D)** Coronal image in a 68-year-old male patient with adenocarcinoma in the left upper lobe. **(E)** Axial image in a 74-year-old male patient with adenocarcinoma in the right upper lobe. **(F)** Axial image in a 68-year-old male patient with squamous cell carcinoma in the right lower lobe.

The lollipop sign was defined as a single, uniformly thickened blood vessel penetrating the middle of the nodule. Specific examples are illustrated in Figure 4 (24).

### Statistical Analysis

All statistical analyses were performed using R software (version 4.5.0; R Foundation for Statistical Computing) and SPSS (version 27.0; IBM). An independent samples *t* test was used to compare normally distributed continuous variables, while the Mann-Whitney *U* test was used in cases where the assumption of normality was not met. Categorical variables were assessed using the Pearson χ^2^ or Fisher exact test. Inter- and intrareader agreements were assessed using κ statistics for categorical variables and intraclass correlation coefficients (ICC) for quantitative variables. κ values were interpreted as follows: slight (<0.20), fair (0.21–0.40), moderate (0.41–0.60), substantial (0.61–0.80), and almost perfect (0.81–1.00) (28). ICC greater than 0.75 was indicative of good or excellent reliability (29). Restricted cubic spline curves were used to assess the potential linear association between IMR and OLNM.

To identify independent CT scan features associated with OLNM, variables were initially screened using univariable logistic regression, followed by multivariable logistic regression with forward stepwise selection based on the Akaike information criterion. A nomogram plot was then developed using the statistically significant variables. The prediction of OLNM using the joint model was validated using the receiver operating characteristic curve and area under the receiver operating characteristic curve (AUC). The optimal critical value was determined using the maximum Youden index. Using the 1000 resamples bootstrap verification method, a calibration curve was plotted to show the relationship between the probability of the actual and predicted outcomes. Additional cross-validation (500 × 10-fold) was performed to obtain the corresponding AUC indexes to avoid overfitting of the model. Decision curve analysis was used to evaluate the clinical validity of the variables. The specificity and cutoff value achieved at 95% sensitivity were determined, because while ensuring that patients who were OLNM-positive were identified as much as possible, it was also crucial to accurately identify patients who were OLNM-negative. Statistical significance for all analyses was set at *P* less than .05.

## Results

### Patient Baseline Characteristics

Among the 329 patients included in this study (median age, 65 years [IQR, 58–70 years]; 168 male, 161 female patients), 40 (12.2%) were currently smoking, 40 (12.2%) had formerly smoked, and the remaining 249 (75.7%) had no smoking history (Table 1). Adenocarcinoma was the most common classification (76.9%; 253 of 329), followed by squamous carcinoma (18.8%; 62 of 329), while the remaining 4.3% (14 of 329) included adenosquamous carcinoma, large cell carcinoma, and carcinoid carcinoma. Patients who were OLNM-positive showed a higher prevalence of the following pathologic features than those who were OLNM-negative: visceral pleural invasion (24 of 73 [32.9%] vs 38 of 256 [14.8%]), spread through air spaces (45 of 73 [61.6%] vs 85 of 256 [33.2%]), lymphovascular invasion (32 of 73 [43.8%] vs 20 of 256 [7.8%]), and perineural invasion (four of 73 [5.5%] vs three of 256 [1.2%]). More than half of the patients had stage IA disease (55.0%; 181 of 329), while only one patient had stage IIIB with group N2 lymph node metastasis. Pathologically confirmed postoperative pathologic T staging was distributed as follows: very few patients (2.4%; eight of 329) had stage T1a disease, none of whom had lymph node metastases; patients with stages T1b, T1c, and T2a had similar percentages (32.5% [107 of 329], 31.3% [103 of 329], and 31.3% [103 of 329], respectively); and approximately 50% of patients with stage T2b (2.4%; eight of 329) had lymph node metastases. Among the total cohort, 73 (22.2%; 73 of 329) patients were OLNM-positive; of these, 35 (47.9%) had N1 nodal metastasis, and 38 (52.1%) had N2 nodal metastasis.

**Table 1:** Clinical and Pathologic Characteristics of Patients and Solid Lung Nodules

Characteristic	OLNM Negative (*n* = 256)	OLNM Positive (*n* = 73)	Total (*n* = 329)
Age (y)	65.0 (59.0–70.0)	64.0 (56.5–69.0)	65.0 (58.0–70.0)
Sex			
Female	125 (48.8)	36 (49.3)	161 (48.9)
Male	131 (51.2)	37 (50.7)	168 (51.1)
Smoking status			
Currently smoking	30 (11.7)	10 (13.7)	40 (12.2)
Formerly smoked	31 (12.1)	9 (12.3)	40 (12.2)
Never smoked	195 (76.2)	54 (74.0)	249 (75.7)
Histology			
Adenocarcinoma	193 (75.4)	60 (82.2)	253 (76.9)
Squamous cell carcinoma	52 (20.3)	10 (13.7)	62 (18.8)
Others	11 (4.3)	3 (4.1)	14 (4.3)
VPI			
Absent	218 (85.2)	49 (67.1)	267 (81.2)
Present	38 (14.8)	24 (32.9)	62 (18.8)
STAS			
Absent	171 (66.8)	28 (38.4)	199 (60.5)
Present	85 (33.2)	45 (61.6)	130 (39.5)
Lymphovascular invasion			
Absent	236 (92.2)	41 (56.2)	277 (84.2)
Present	20 (7.8)	32 (43.8)	52 (15.8)
Perineural invasion			
Absent	253 (98.8)	69 (94.5)	322 (97.9)
Present	3 (1.2)	4 (5.5)	7 (2.1)
Clinical stage			
IA	181 (70.7)	0 (0)	181 (55.0)
IB	71 (27.7)	0 (0)	71 (21.6)
IIA	4 (1.6)	0 (0)	4 (1.2)
IIB	0 (0)	35 (47.9)	35 (10.6)
IIIA	0 (0)	37 (50.7)	37 (11.2)
IIIB	0 (0)	1 (1.4)	1 (0.3)
Pathologic T stage			
T1a	8 (3.1)	0 (0)	8 (2.4)
T1b	95 (37.1)	12 (16.4)	107 (32.5)
T1c	78 (30.5)	25 (34.2)	103 (31.3)
T2a	71 (27.7)	32 (43.8)	103 (31.3)
T2b	4 (1.6)	4 (5.5)	8 (2.4)
Lymph node involvement			
N0	256 (100.0)	0 (0.0)	256 (77.8)
N1	0 (0.0)	35 (47.9)	35 (10.6)
N2	0 (0.0)	38 (52.1)	38 (11.6)

Note.—Categorical values are presented as number of nodules, with percentages in parentheses. Continuous variables are presented as medians, with IQRs in parentheses. OLNM = occult lymph node metastasis, STAS = spread through air spaces, VPI = visceral pleural invasion.

### Intra- and Interreader Agreement of CT Predictors

The consistency of several characteristics, including IMR, OMR, inner margin location, tumor size, lollipop sign, satellite lesion, tumor-pleura relationship, contact length with pleura, contact length with pleura ratio, and pleural adhesion, was investigated using inter- and intrareader repeated assessments (Table S1). The results of these assessments showed consistency within one reader with a consistently high Cohen κ (>0.83) and ICC (>0.79) for all characteristics. Among two readers, all characteristics had a strong or good consistency (κ = 0.71–0.93; ICC = 0.71–0.89). Figure S1 illustrates the correlation between the two repeated assessments of IMR and OMR at 2-week intervals.

### CT Scan Features of Solid NSCLCs for Predicting OLNM

Table 2 lists the main relevant CT scan features with and without OLNM. Detailed noncontrast-enhanced and contrast-enhanced CT scan features are presented in Tables S2 and S3. The frequency of right upper lung cancer was 31.3% (103 of 329). Compared with those without OLNM, nodules with OLNM had a larger tumor size (median, 26 mm vs 22 mm; *P* < .001), smaller IMR (median, 0.48 vs 0.58; *P* < .001), longer contact length with pleura (median, 7.0 vs 0.0 mm; *P* < .001), and a greater contact length with pleura ratio (median, 0.34 vs 0.00; *P* < .001). However, there was no evidence of a difference in OMR for identification of OLNM (*P* = .63). Nodules with or without OLNM differed in their inner margin location (*P* < .001). Tumor-pleura relationship types II and III were more common among patients with OLNM (35.6% [26 of 73] vs 29.7% [76 of 256], and 56.2% [41 of 73] vs 28.5% [73 of 256], respectively; *P* < .001) than among those without. Multiple pleural adhesions (47.9% [35 of 73] vs 31.6% [81 of 256]; *P* = .002), the lollipop sign (65.8% [48 of 73] vs 42.2% [108 of 256]; *P* < .001), and satellite lesions (79.5% [58 of 73] vs 66.0% [169 of 256]; *P* = .03) were more frequent among patients with OLNM than among those without.

**Table 2:** Characteristics of Solid Lung Nodules

Characteristic	OLNM Negative (*n* = 256)	OLNM Positive (*n* = 73)	Total (*n* = 329)	*P* Value
Nodule location				.97
Right upper lobe	81 (31.6)	22 (30.1)	103 (31.3)	
Right middle lobe	12 (4.7)	5 (6.8)	17 (5.2)	
Right lower lobe	48 (18.8)	13 (17.8)	61 (18.5)	
Left upper lobe	61 (23.8)	18 (24.7)	79 (24.0)	
Left lower lobe	54 (21.1)	15 (20.5)	69 (21.0)	
Size (mm)	22 (16–27)	26 (20–32)	22 (17–28)	<.001*
Inner margin ratio	0.58 (0.44–0.73)	0.48 (0.33–0.62)	0.57 (0.42–0.71)	<.001*
Outer margin ratio	0.90 (0.73–0.96)	0.88 (0.70–1.00)	0.89 (0.73–0.97)	.63
Inner margin location				<.001*
Medial	27 (10.5)	20 (27.4)	47 (14.3)	
Intermedius	140 (54.7)	42 (57.5)	182 (55.3)	
Lateral	89 (34.8)	11 (15.1)	100 (30.4)	
Shape				.09
Round	81 (31.6)	33 (45.2)	114 (34.7)	
Oval	38 (14.8)	7 (9.6)	45 (13.7)	
Irregular	137 (53.5)	33 (45.2)	170 (51.7)	
Lobulation				.58
Absent	29 (11.3)	10 (13.7)	39 (11.9)	
Present	227 (88.7)	63 (86.3)	290 (88.1)	
Spiculation				.55
Absent	123 (48.0)	38 (52.1)	161 (48.9)	
Present	133 (52.0)	35 (47.9)	168 (51.1)	
Bronchus				.53
Cutoff	149 (58.2)	42 (57.5)	191 (58.1)	
Traction	89 (34.8)	23 (31.5)	112 (34.0)	
Wall thickening	18 (7.0)	8 (11.0)	26 (7.9)	
Tumor-pleura relationship				<.001*
I	107 (41.8)	6 (8.2)	113 (34.3)	
II	76 (29.7)	26 (35.6)	102 (31.0)	
III	73 (28.5)	41 (56.2)	114 (34.7)	
Pleural adhesion				.002*
Absent	54 (21.1)	4 (5.5)	58 (17.6)	
Single	121 (47.3)	34 (46.6)	155 (47.1)	
Multiple	81 (31.6)	35 (47.9)	116 (35.3)	
Lollipop sign				<.001*
Absent	148 (57.8)	25 (34.2)	173 (52.6)	
Present	108 (42.2)	48 (65.8)	156 (47.4)	

Note.—Categorical values are presented as numbers of nodules, with percentages in parentheses. Continuous variables are presented as medians, with IQRs in parentheses. OLNM = occult lymph node metastasis.

* *P* values were calculated using the Mann-Whitney *U* test for continuous variables and the Pearson χ^2^ and Fisher exact tests for categorical variables.

**Figure 5:**
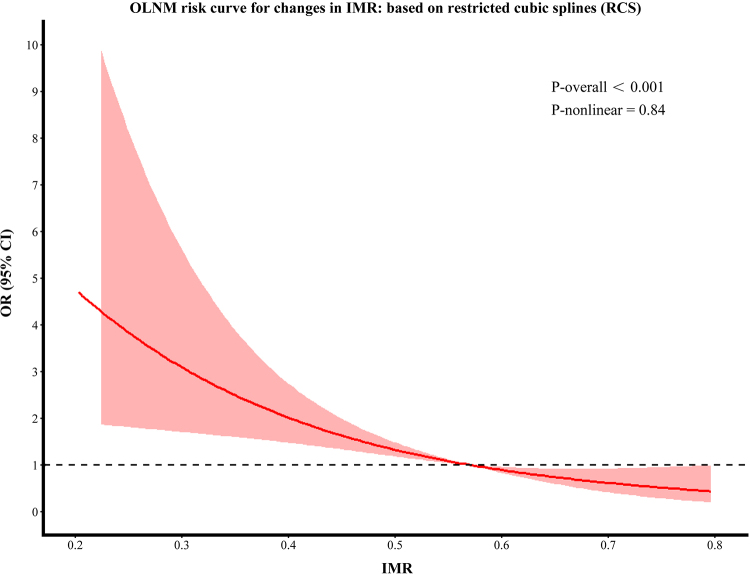
Occult lymph node metastasis (OLNM) risk curve for changes in inner margin ratio (IMR). In the restricted cubic splines (RCS) regression, adjustments were made for lollipop sign and tumor-pleura relationship. OR = odds ratio.

After adjusting for potential confounders, restricted cubic spline analysis was performed, which showed an association between IMR and OLNM (*P*-overall < .001; *P*-nonlinear = .84) (Fig 5), with larger IMR associated with a progressively lower risk of OLNM.

### Independent Predictors for OLNM and Model Performance

**Figure 6:**
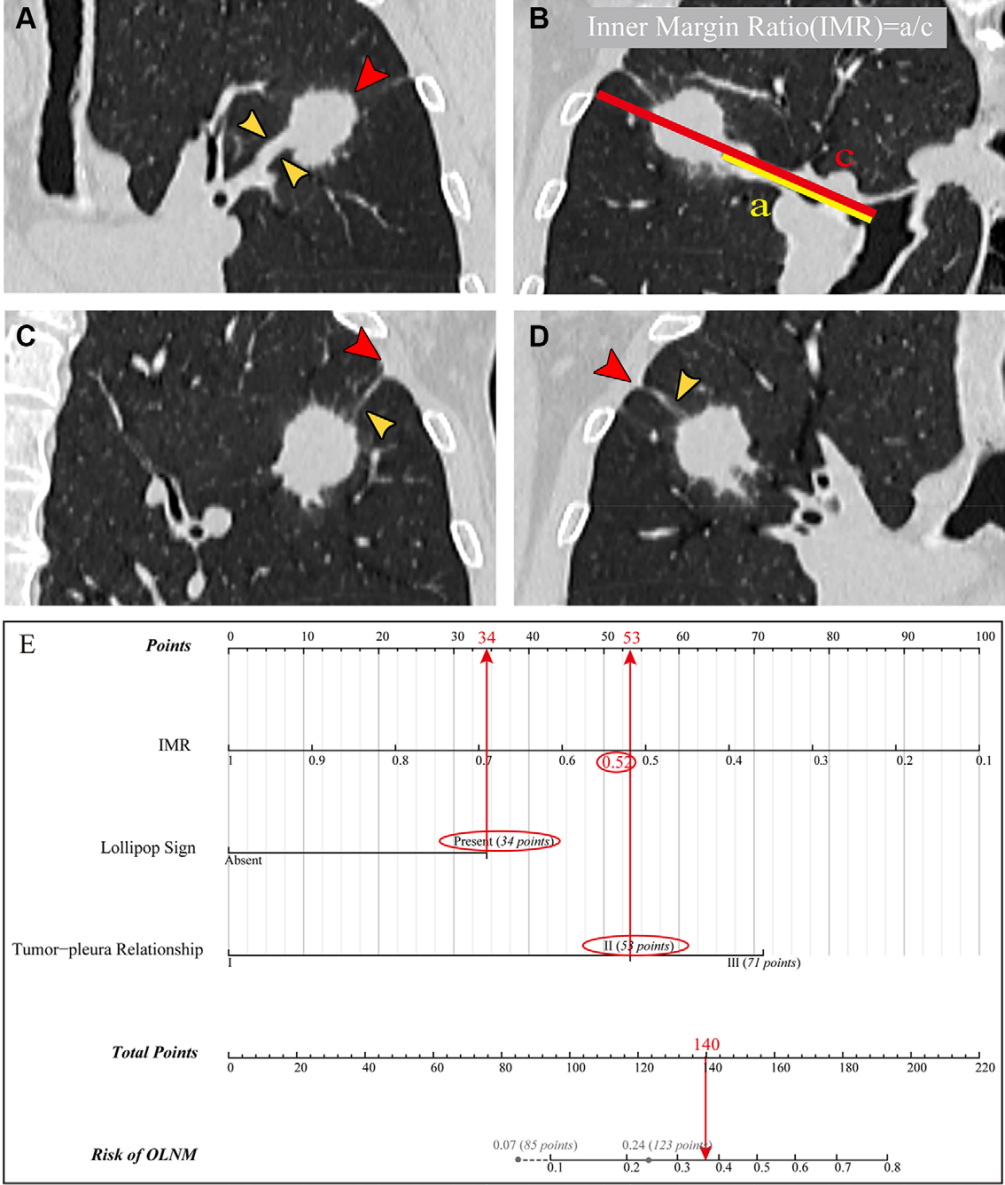
The key CT scan features in a 74-year-old male patient are shown in **(A–D)**; postoperative pathology confirmed the patient had N1 lymph node metastasis. The application of nomogram based on inner margin ratio (IMR), lollipop sign, and tumor-pleura relationship is shown in **(E)**. When the model reached the highest area under the receiver operating characteristic curve of 0.81, the risk of occult lymph node metastasis (OLNM) was 0.24. When the model reached a sensitivity of 95%, the risk of OLNM was 0.07. **(A)** Lollipop sign: the red arrowhead indicates the lung nodule, and the yellow arrowheads indicate the single blood vessel entry. **(B)** IMR was 50.1/97.1 mm = 0.52. **(C, D)** Tumor-pleura relationship type II: the red arrowheads indicate pleural indentation, and the yellow arrowheads indicate the linear pleural tag. **(E)** Total score of nomogram was 140 points. Risk of OLNM was 0.36.

**Figure 7:**
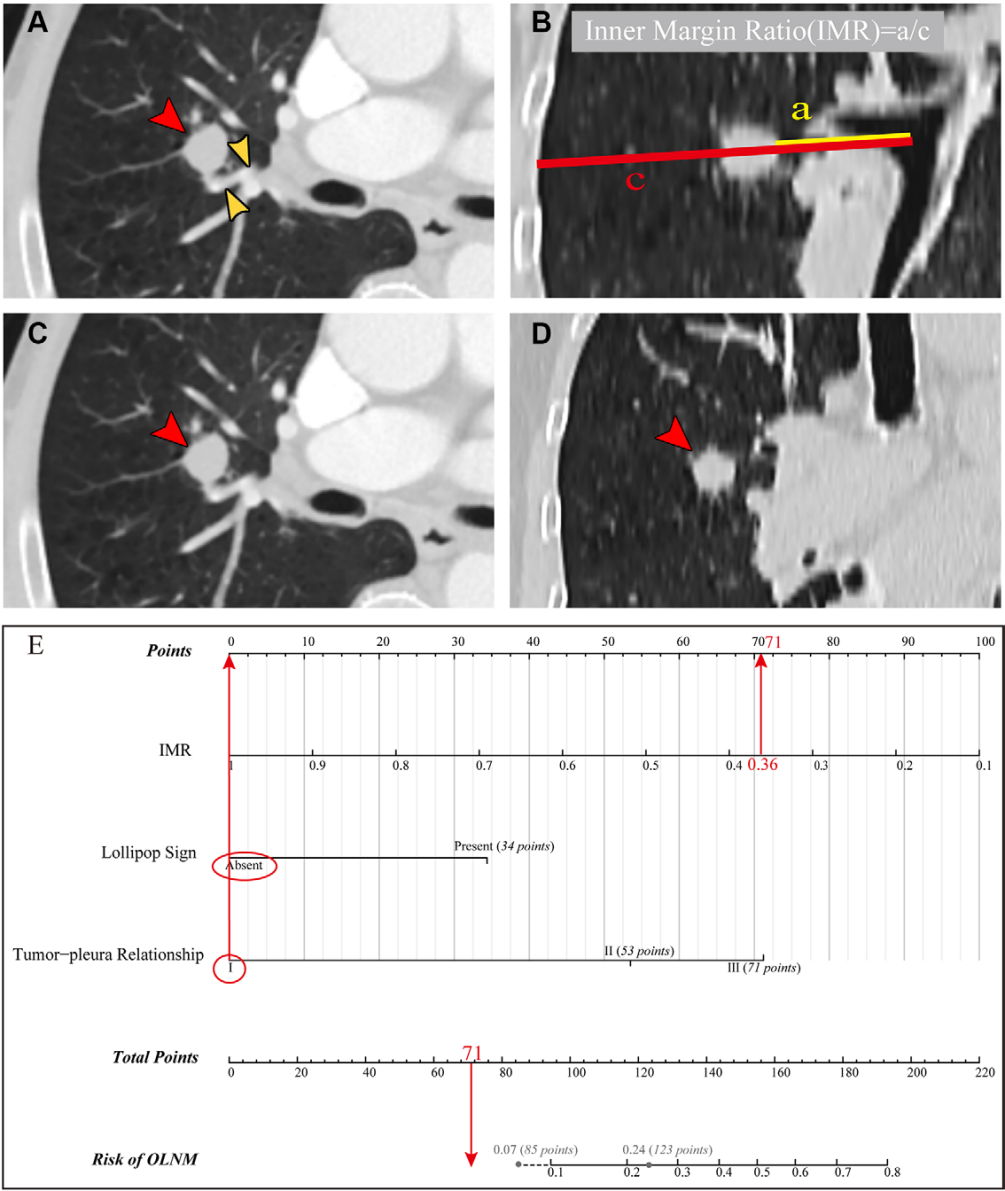
The key image features in a 77-year-old male patient are shown in **(A–D)** CT images; postoperative pathology confirmed the patient did not have lymph node metastasis. The application of nomogram based on inner margin ratio (IMR), lollipop sign, and tumor-pleura relationship is shown in **(E)**. When the model reached the highest area under the receiver operating characteristic curve of 0.81, the risk of occult lymph node metastasis (OLNM) was 0.24. When the model reached a sensitivity of 95%, the risk of OLNM was 0.07. **(A)** No lollipop sign: the red arrowhead indicates the lung nodule, and the yellow arrowheads indicate the blood vessel had not penetrated the nodule. **(B)** IMR was 29.6 mm/82.2 mm = 0.36. **(C, D)** Tumor-pleura relationship type I: the red arrowheads indicate the lung nodule had no contact with pleura. **(E)** Total score of nomogram was 71 points. Risk of OLNM was less than 0.07.


Multivariable logistic regression (Table 3) identified IMR (odds ratio [OR], 0.02; 95% CI: 0.00, 0.10; *P* < .001), the lollipop sign (OR, 3.48; 95% CI: 1.87, 6.49; *P* < .001), and tumor-pleura relationship type II (OR, 6.95; 95% CI: 2.62, 18.44; *P* < .001) and type III (OR, 13.27; 95% CI: 5.11, 34.45; *P* < .001) as independent predictors of OLNM. A nomogram was constructed based on these findings, achieving an AUC of 0.81 (95% CI: 0.76, 0.87), sensitivity of 78.1%, specificity of 73.4%, positive predictive value of 45.2%, negative predictive value of 92.1%, maximal Youden index of 0.51, and cutoff value of 0.24 (Fig S2). Figures 6 and 7, along with Figure S3, demonstrate the clinical application of the predictive model via a nomogram for individualized risk assessment. The binary logistic regression equation was as follows:

**Table 3:** Univariable and Multivariable Logistic Regression Analyses for Predicting OLNM in Solid Lung Nodules

Characteristic	Univariable Logistic Regression Analysis		Multivariable Logistic Regression Analysis	
	OR	*P* Value	OR	*P* Value
Size (mm)	1.07 (1.04, 1.11)	<.001		NA
Inner margin ratio	0.05 (0.01, 0.19)	<.001	0.02 (0.00, 0.10)	<.001*
Inner margin location				
Medial	6.00 (2.56, 14.06)	<.001		
Intermedius	2.43 (1.19, 4.96)	.02		
Lateral	Reference			
Satellite lesion (yes)	1.99 (1.07, 3.72)	.03		NA
Lollipop sign (yes)	2.63 (1.53, 4.53)	<.001	3.48 (1.87, 6.49)	<.001*
Tumor-pleura relationship				
I	Reference			
II	6.10 (2.40, 15.54)	<.001	6.95 (2.62, 18.44)	<.001*
III	10.02 (4.04, 24.81)	<.001	13.27 (5.11, 34.45)	<.001*
Contact length with pleura (mm)	1.05 (1.02, 1.07)	<.001		NA
Contact length with pleura ratio	2.71 (1.48, 4.97)	<.001		NA
Pleural adhesion				
Absent	Reference			
Single	3.79 (1.28, 11.22)	.02		
Multiple	5.83 (1.96, 17.36)	.002		

Note.—Data in parentheses are 95% CIs. OLNM = occult lymph node metastasis, OR = odds ratio, NA = not applicable.

* *P* value was calculated with multivariable logistic regression analysis using the forward stepwise method.

Logit(P)=-1.588 + 1.248 × x1 + 1.942 × x2 + 2.585 × x3-4.033 × x4,

where* x*1 is the lollipop sign,* x*2 is the tumor-pleura relationship type II,* x*3 is the tumor-pleura relationship type III, and* x*4 is the IMR.

### Model Correction and Evaluation

Calibration curves were plotted using 1000 resample bootstrap verification (Fig S4), which showed that the predicted and actual values were in good agreement. To avoid overfitting, a 500 × 10-fold internal cross-validation was performed, with the AUC remaining stable at 0.81. Additionally, a decision curve was constructed to validate the usefulness of the model for clinical use (Fig S5).

Table 4 demonstrates the differences in IMR, the lollipop sign, and tumor-pleura relationship and all other meaningful one-way logistic regression imaging features between the N1 and N2 (*n* = 73) subgroups. IMR, the lollipop sign, and the tumor-pleura relationship (Figs S6 and S7) showed no evidence of a difference between the subgroups (*P* > .05).

**Table 4:** Comparison of CT Predictors of OLNM in Solid Lung Nodules by N1 and N2 Stage

Characteristic	N1 (*n* = 35)	N2 (*n* = 38)	Total (*n* = 73)	*P* Value
Size (mm)	26 (20–34)	25 (20–31)	26 (20–32)	.49
Inner margin ratio	0.37 (0.26–0.63)	0.50 (0.37–0.62)	0.48 (0.33–0.62)	.15
Inner margin location				.04*
Medial	14 (40.0)	6 (15.8)	20 (27.4)	
Intermedius	15 (42.9)	27 (71.1)	42 (57.5)	
Lateral	6 (17.1)	5 (13.2)	11 (15.1)	
Satellite lesion				.06
Absent	4 (11.4)	11 (28.9)	15 (20.5)	
Present	31 (88.6)	27 (71.1)	58 (79.5)	
Lollipop sign				.63
Absent	11 (31.4)	14 (36.8)	25 (34.2)	
Present	24 (68.6)	24 (63.2)	48 (65.8)	
Tumor-pleura relationship				.77
I	3 (8.6)	3 (7.9)	6 (8.2)	
II	11 (31.4)	15 (39.5)	26 (35.6)	
III	21 (60.0)	20 (52.6)	41 (56.2)	
Contact length with pleura (mm)	11.0 (0.0–25.0)	5.5 (0.0–18.3)	7.0 (0.0–20.5)	.38
Contact length with pleura ratio	0.42 (0.00–0.75)	0.25 (0.00–0.69)	0.34 (0.00–0.75)	.44
Pleural adhesion				.94
Absent	2 (5.7)	2 (5.3)	4 (5.5)	
Single	17 (48.6)	17 (44.7)	34 (46.6)	
Multiple	16 (45.7)	19 (50.0)	35 (47.9)	

Note.—Categorical values are presented as numbers of nodules, with percentages in parentheses. Continuous variables are presented as medians, with IQRs in parentheses. OLNM = occult lymph node metastasis.

* *P* values were calculated using the Mann-Whitney *U* test for continuous variables and the Pearson χ^2^ and Fisher exact tests for categorical variables.

### Patterns in Patients Negative for OLNM

**Figure 8:**
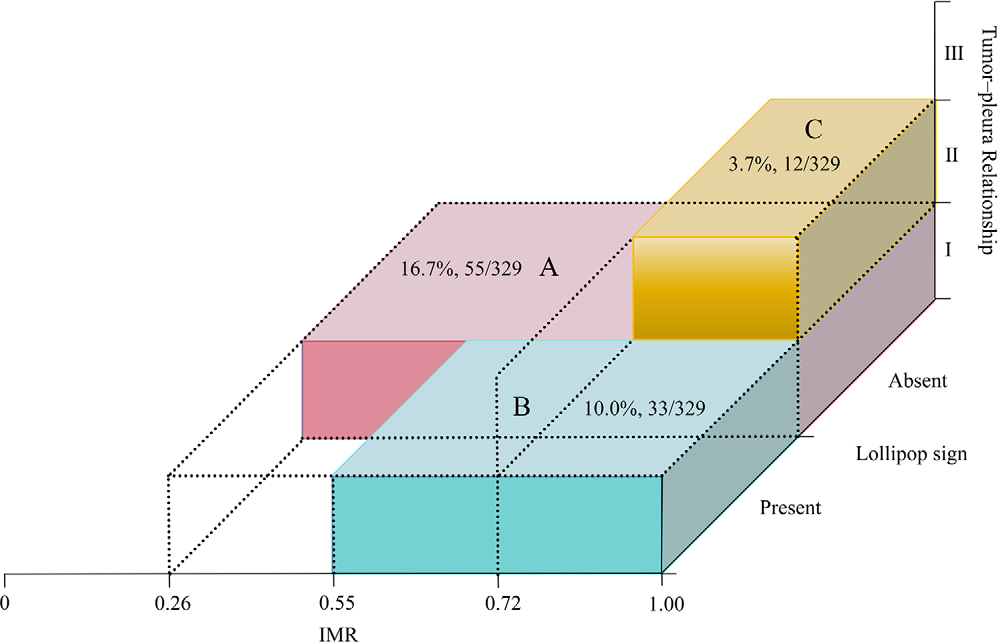
Three-dimensional pattern diagram of patients who did not have occult lymph node metastasis. The x-axis represents inner margin ratio (IMR), the y-axis represents lollipop sign, and the z-axis represents tumor-pleura relationship. The pink rectangle indicates pattern A, the blue rectangle indicates pattern B, and the yellow rectangle indicates pattern C.

With a model sensitivity of 95%, the specificity was 39.1% and the cutoff value was 0.07. To enhance the clinical utility of the model, we identified three patterns among the patients who were OLNM-negative (39.1% specificity) based on tumor-pleura relationship type, lollipop sign status, and IMR. These patterns are visualized in a three-dimensional chart (Fig 8) and summarized as follows: *(a)* tumor-pleura relationship type I, without a lollipop sign, and an IMR of 0.26 or more; *(b)* tumor-pleura relationship type I, with a lollipop sign, and an IMR of 0.55 or more; and *(c)* tumor-pleura relationship type II, without a lollipop sign, and an IMR of 0.72 or more.

## Discussion

OLNM is a crucial prognostic indicator of NSCLC. In this retrospective review of 329 solid NSCLC nodules, the positivity rate for OLNM was 22.2% (73 of 329). Our results showed that preoperative contrast-enhanced CT-derived IMR, lollipop sign, and tumor-pleura relationship types II and III were excellent predictors of OLNM. Additionally, the resulting model had an AUC of 0.81, with a sensitivity of 78.1% and specificity of 73.4%.

The American College of Chest Physicians guidelines define tumors located in the medial one-third of the chest as central (30), whereas the National Comprehensive Cancer Network guidelines define those located in the medial two-thirds of the chest as central (31). Sanz-Santos et al selected the largest level of the tumor in the axial CT plane, plotted the median and outer margin plumb lines of the thorax, and defined the tumor in the inner one-third of the axial vertical line as the central tumor, which predicted the N1 group of lymph nodes but failed to predict the N2 group (32). Kawamoto et al concentrated on the nodules in the lower lobes of both lungs and found that IMR, determined in the axial plane, effectively predicted N2 group lymph node metastasis when the tumors were categorized into the medial (IMR ≤0.5) and lateral (IMR >0.5) groups (33). Most of the previous studies considered the IMR on axial images and classified it using thresholds, such as one-third or one-half; however, considering that tumors are three-dimensional nodules, we chose to calculate IMR from multiplanar reconstruction images and observed a significant linear relationship between IMR and OLNM when measured from the bronchial entry of the lung lobe. IMR itself was more meaningful than the three- or two-category classifications in predicting OLNM (including N1 and N2). However, we did not observe a correlation between OMR and OLNM, which is consistent with the findings of Siwachat et al (34).

The lollipop sign is thought to be associated with hepatic epithelioid hemangioendothelioma (35). However, Sun and Li et al (24) found that it could be used to predict angiolymphatic invasion in NSCLCs measuring less than 30 mm; however, whether the lollipop sign can be used to predict OLNM had yet to be confirmed. While reviewing patient images, we found that OLNM-positive nodules were often accompanied by a thickened blood vessel, indicating that the lollipop sign was an excellent predictor of OLNM. Although routine vessels emanate from the hilum taper, thickened vessels penetrating the nodule may be an indirect manifestation of tumor infiltration of the vasculature/lymphatics. We included additional enhancement features, such as bronchovascular bundle thickening, arterial or venous enhancement degree, rate, and mode, to determine the correlation between tumor blood supply and OLNM, as previous studies have not focused on these features. Unfortunately, there was no evidence of a difference in any of the aforementioned features for predicting OLNM, which is consistent with the findings of Zhao et al (36).

Previous studies confirmed that multiple pleural tags and adhesions are important predictors of visceral pleural invasion (37) and are more likely to lead to lymph node metastasis (38,39). Therefore, we focused on the tumor-pleura relationship, which was classified into three types. We found that patients with OLNM primarily had types II and III relationships, whereas type I was rare. Zhang et al (25) observed that combining tumor-pleura relationship types II and III had predictive value for OLNM in peripheral clinical stage IA solid adenocarcinoma. Our findings indicate that when analyzed as distinct categories, types II and III both served as strong and independent predictors of OLNM compared with type I, with type III exhibiting a higher predictive power. This discrepancy may be explained by the relatively small type II tumor-pleura relationship sample size (*n* = 19) from Zhang et al’s studies, whereas our sample size was larger (*n* = 102). Other pleura-related factors such as pleura contact length, ratio, and number of adhesions differed between patients with and without OLNM, although there was no evidence of a difference in the multivariable logistic regression analysis.

The primary contrast-enhanced CT scan features indicative of OLNM—IMR, lollipop sign, and tumor-pleura relationship—are reliable and easily obtainable through the standard preoperative imaging. Furthermore, a phase III study (18) found that selective LND improved perioperative outcomes compared with systematic LND. The findings of our study indicated that 77.8% (256 of 329) of patients underwent unnecessary systematic LND; therefore, this study aimed to find imaging features of patients who are OLNM-negative to ensure they avoid unnecessary surgery. These findings are expected to be viable for use in the clinical setting.

This study had some limitations. First, this was a single-center, retrospective study. Second, the positive predictive value of this model was not ideal, possibly because the OLNM positivity rate among the study cohort was relatively low, although close to previous findings. Third, considering the complexity of the lymphatic drainage system in the lungs, further studies are needed to investigate the frequency of lymph node metastasis in different lobes of the lungs and other predictive indicators. Fourth, given that current guidelines acknowledge the role of PET/CT in the preoperative assessment of lymph node status, this modality should be incorporated into future studies to validate and extend our findings. Fifth, an inherent limitation of our model was its dependence on statistical significance for the selection of variables, which is constrained by the statistical power to identify weaker but potentially important clinical associations. Future work will utilize machine learning approaches to validate and refine our model. Finally, as this study lacked an external validation group, cross-validation and bootstrap methods were performed to avoid overfitting, and the efficacy of the obtained model was close to that of the original model. In the future, we will expand the sample size to validate and optimize the diagnostic efficacy of this model and further study the effective factors among the various subgroups.

In conclusion, preoperative contrast-enhanced CT scan features, particularly IMR, the lollipop sign, and tumor-pleura relationship, were independent predictors of OLNM among patients with early-stage solid NSCLC and demonstrated predictive value for both N1 and N2 nodal groups. In the future, larger multi-institutional studies are needed to establish more accurate models for predicting OLNM in these patients.

## Supplemental Files

Tables S1-S3, Figures S1-S7

Conflicts of Interest
