# Supplementary material for: Preoperative Contrast-enhanced CT Features Associated with Occult Lymph Node Metastasis in Early-Stage Solid Non–Small Cell Lung Cancer
Source: Radiol Imaging Cancer. 2026 Mar 6;8(2):e250448. doi: 10.1148/rycan.250448 (PMC13036687; doi:10.1148/rycan.250448)
Supplement: Tables S1-S3, Figures S1-S7 [file rycan250448suppa1.pdf]

Supplemental materials undergo peer review but are not copyedited, and may include typographical errors.

©RSNA, 2026
10.1148/rycan.250448

Table S1: Intra- and Interreader Agreement on CT Characteristics Predicting OLNM (*n* = 329)

Characteristic	Intrareader Agreement	Interreader Agreement
Inner margin location	0.96 (0.89, 1.00)	0.93 (0.82, 1.00)
Inner margin ratio	0.96 (0.95, 0.98)	0.89 (0.84, 0.93)
Outer margin ratio	0.93 (0.88, 0.98)	0.89 (0.82, 0.96)
Size (mm)	0.90 (0.86, 0.93)	0.82 (0.76, 0.89)
Lollipop sign	0.88 (0.72, 1.00)	0.75 (0.53, 0.97)
Satellite lesion	0.83 (0.64, 1.00)	0.71 (0.48, 0.95)
Tumor-pleura relationship	0.90 (0.78, 1.00)	0.82 (0.68, 0.97)
Contact length with pleura (mm)	0.89 (0.80, 0.97)	0.78 (0.62, 0.95)
Contact length with pleura ratio	0.79 (0.65, 0.92)	0.71 (0.57, 0.86)
Pleural adhesion	0.87 (0.73, 1.00)	0.78 (0.59, 0.96)

Note.—Categorical variables were evaluated with Cohens Kappa coefficient, while continuous variables were assessed using the intraclass correlation coefficient. Data in parentheses are 95% CIs. IMR = inner margin ratio, OLNM = occult lymph node metastasis, OMR = outer margin ratio.

Supplemental materials undergo peer review but are not copyedited, and may include typographical errors.

Table S2: Noncontrast-Enhanced CT Radiologic Characteristics of Solid Lung Nodules

Characteristic	OLNM Negative (n = 256)	OLNM Positive (n = 73)	Total (n = 329)	P Value
Nodule location				.97
Right upper lobe	81 (31.6)	22 (30.1)	103 (31.3)	
Right middle lobe	12 (4.7)	5 (6.8)	17 (5.2)	
Right lower lobe	48 (18.8)	13 (17.8)	61 (18.5)	
Left upper lobe	61 (23.8)	18 (24.7)	79 (24.0)	
Left lower lobe	54 (21.1)	15 (20.5)	69 (21.0)	
Size (mm)	22 (16, 27)	26 (20, 32)	22 (17, 28)	< .001*
Inner margin ratio	0.58 (0.44, 0.73)	0.48 (0.33, 0.62)	0.57 (0.42, 0.71)	< .001*
Outer margin ratio	0.90 (0.73, 0.96)	0.88 (0.70, 1.00)	0.89 (0.73, 0.97)	.63
Inner margin location				< .001*
Medial	27 (10.5)	20 (27.4)	47 (14.3)	
Intermedius	140 (54.7)	42 (57.5)	182 (55.3)	
Lateral	89 (34.8)	11 (15.1)	100 (30.4)	
Lung surface margin				.84
Smooth	14 (5.5)	5 (6.8)	19 (5.8)	
Clear	180 (70.3)	49 (67.1)	229 (69.6)	
Blurry	62 (24.2)	19 (26.0)	81 (24.6)	
Shape				.09
Round	81 (31.6)	33 (45.2)	114 (34.7)	
Oval	38 (14.8)	7 (9.6)	45 (13.7)	
Irregular	137 (53.5)	33 (45.2)	170 (51.7)	
Lobulation				.58
Absent	29 (11.3)	10 (13.7)	39 (11.9)	
Present	227 (88.7)	63 (86.3)	290 (88.1)	
Spiculation				.55
Absent	123 (48.0)	38 (52.1)	161 (48.9)	
Present	133 (52.0)	35 (47.9)	168 (51.1)	
Calcification				.24
Absent	246 (96.1)	72 (98.6)	318 (96.7)	
Present	10 (3,9)	1 (1.4)	11 (3.3)	
Bronchus				.53
Cut-off	149 (58.2)	42 (57.5)	191 (58.1)	
Traction	89 (34.8)	23 (31.5)	112 (34.0)	
Wall thickening	18 (7.0)	8 (11.0)	26 (7.9)	
Air Bronchogram				.92
Absent	149 (58.2)	42 (57.5)	191 (58.1)	
Present	107 (41.8)	31 (42.5)	138 (41.9)	
Cystic airspace				.58
Absent	210 (82.0)	59 (80.8)	269 (81.8)	
Vacuole	44 (17.2)	12 (16.4)	56 (17.0)	

Supplemental materials undergo peer review but are not copyedited, and may include typographical errors.

Cavity	2 (0.8)	2 (2.7)	4 (1.2)	
Peripheral GGO				.21
Absent	225 (87.9)	60 (82.2)	285 (86.6)	
Present	31 (12.1)	13 (17.8)	44 (13.4)	
Satellite lesion				.03*
Absent	87 (34.0)	15 (20.5)	102 (31.0)	
Present	169 (66.0)	58 (79.5)	227 (69.0)	
Tumor-pleura relationship				< .001*
I	107 (41.8)	6 (8.2)	113 (34.3)	
II	76 (29.7)	26 (35.6)	102 (31.0)	
III	73 (28.5)	41 (56.2)	114 (34.7)	
Contact length with pleura (mm)	0.0 (0.0, 12.0)	7.0 (0.0, 20.5)	0.0 (0.0, 14.0)	< .001*
Contact length with pleura Ratio	0.00 (0.00, 0.55)	0.34 (0.00, 0.75)	0.00 (0.00, 0.63)	< .001*
Pleural adhesion				.002*
Absent	54 (21.1)	4 (5.5)	58 (17.6)	
Single	121 (47.3)	34 (46.6)	155 (47.1)	
Multiple	81 (31.6)	35 (47.9)	116 (35.3)	
Tumor side pleural effusion				.56
Absent	249 (97.3)	70 (95.9)	319 (97.0)	
Present	7 (2.7)	3 (4.1)	10 (3.0)	
Nontumor side pleural effusion				.85
Absent	250 (97.7)	71 (97.3)	321 (97.6)	
Present	6 (2.3)	2 (2.7)	8 (2.4)	
Pericardial effusion				.35
Absent	253 (98.8)	73 (100)	326 (99.1)	
Present	3 (1.2)	0 (0)	3 (0.9)	

Note.— Categorical values are presented as numbers of nodules, with percentages in parentheses. Continuous variables are presented as medians, with interquartile ranges (IQRs) in parentheses. **P* values were calculated with the Mann-Whitney *U* test for continuous variables and Pearson $\chi 2$ and Fisher exact tests for categorical variables. OLNM = occult lymph node metastasis. GGO = ground-glass opacification.

Supplemental materials undergo peer review but are not copyedited, and may include typographical errors.

Table S3: Contrast-Enhanced CT Radiologic Characteristics of Solid Lung Nodules

Characteristic	OLNM Negative (*n* = 256)	OLNM Positive (*n* = 73)	Total (*n* = 329)	*P* Value
Lollipop sign				< .001*
Absent	148 (57.8)	25 (34.2)	173 (52.6)	
Present	108 (42.2)	48 (65.8)	156 (47.4)	
Bronchovascular Bundle Thickening				.08
Absent	235 (91.8)	62 (84.9)	297 (90.3)	
Present	21 (8.2)	11 (15.1)	32 (9.7)	
Arterial enhancement degree	20 (14, 32)	20 (12, 33)	20 (13, 32)	.45
Venous enhancement degree	28 (19, 36)	29 (21, 36)	28 (20, 36)	.66
Arterial Enhancement Rate	0.85 (0.48, 1.33)	0.80 (0.30, 1.32)	0.85 (0.44, 1.33)	.10
Venous Enhancement Rate	1.00 (0.63, 1.50)	0.89 (0.62, 1.37)	1.00 (0.63, 1.48)	.12
Enhancement Mode				.46
Homogeneous	149 (58.2)	46 (63.0)	195 (59.3)	
Nonhomogeneous	107 (41.8)	27 (37.0)	134 (40.7)	

Note.—Categorical values are presented as numbers of nodules, with percentages in parentheses. Continuous variables are presented as medians, with IQRs in parentheses.
**P* values were calculated with the Mann–Whitney *U* test for continuous variables and Pearson $\chi 2$ and Fisher exact tests for categorical variables. OLNM = occult lymph node metastasis.

Supplemental materials undergo peer review but are not copyedited, and may include typographical errors.

Supplemental Figure Legends

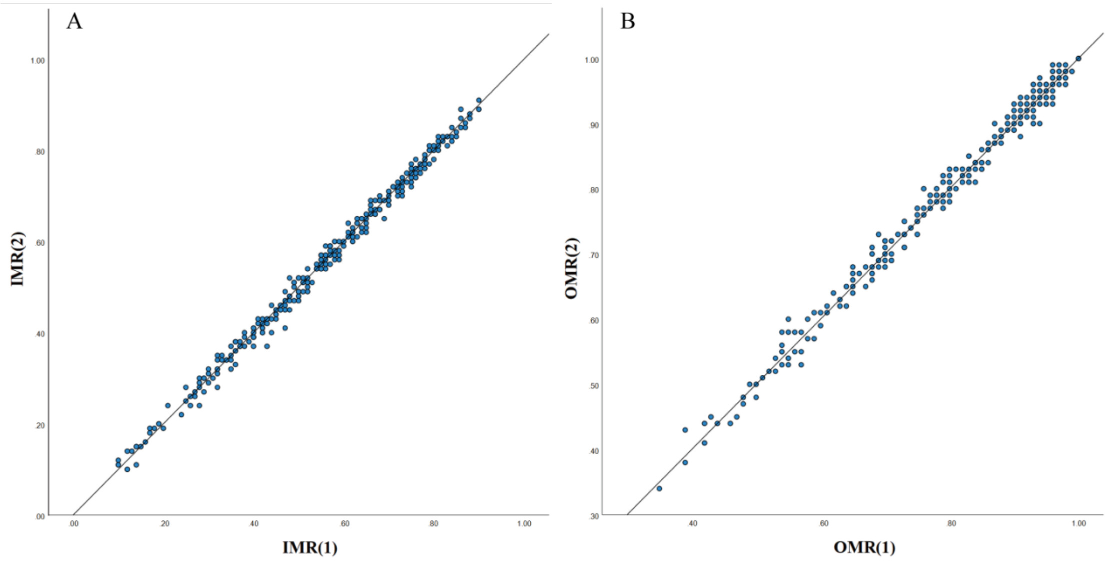

Figure S1: Scatter plot displaying the correlation between two repetitions of IMR **(A)** and OMR **(B)** with a 2-week interval. IMR = inner margin ratio. OMR = outer margin ratio.

Supplemental materials undergo peer review but are not copyedited, and may include typographical errors.

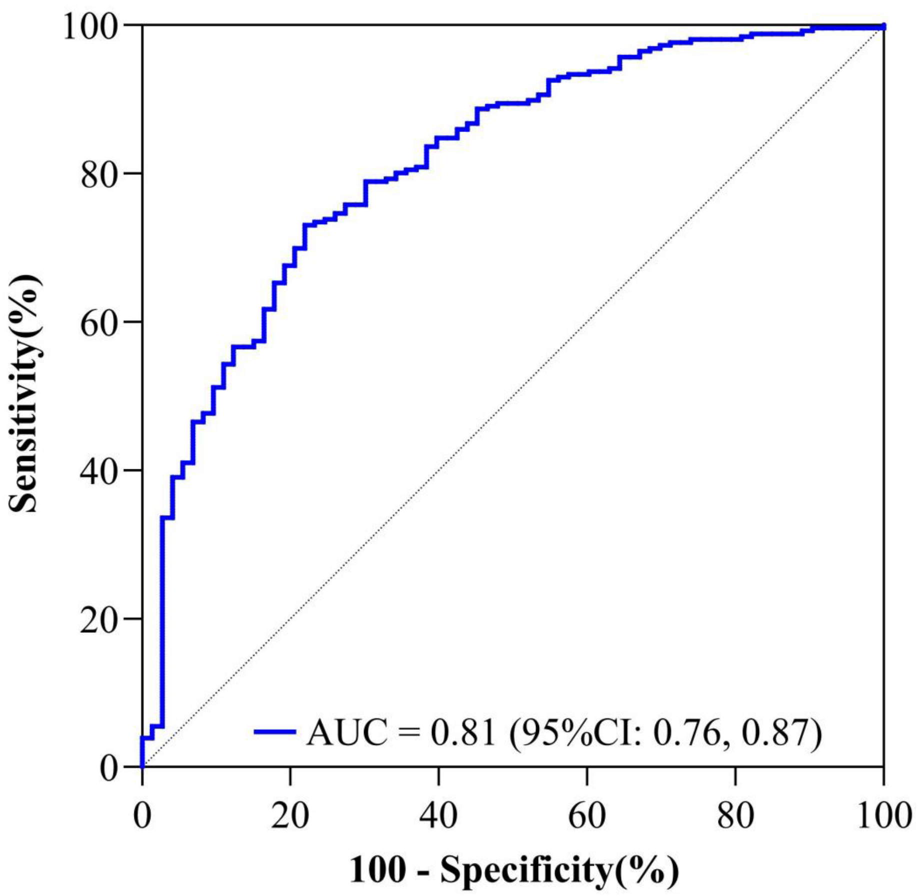

Figure S2: ROC curves of nomogram model to predict OLNM. ROC = receiver operating characteristic. AUC = area under the receiver operating characteristic curve, OLNM = occult lymph node metastasis.

Supplemental materials undergo peer review but are not copyedited, and may include typographical errors.

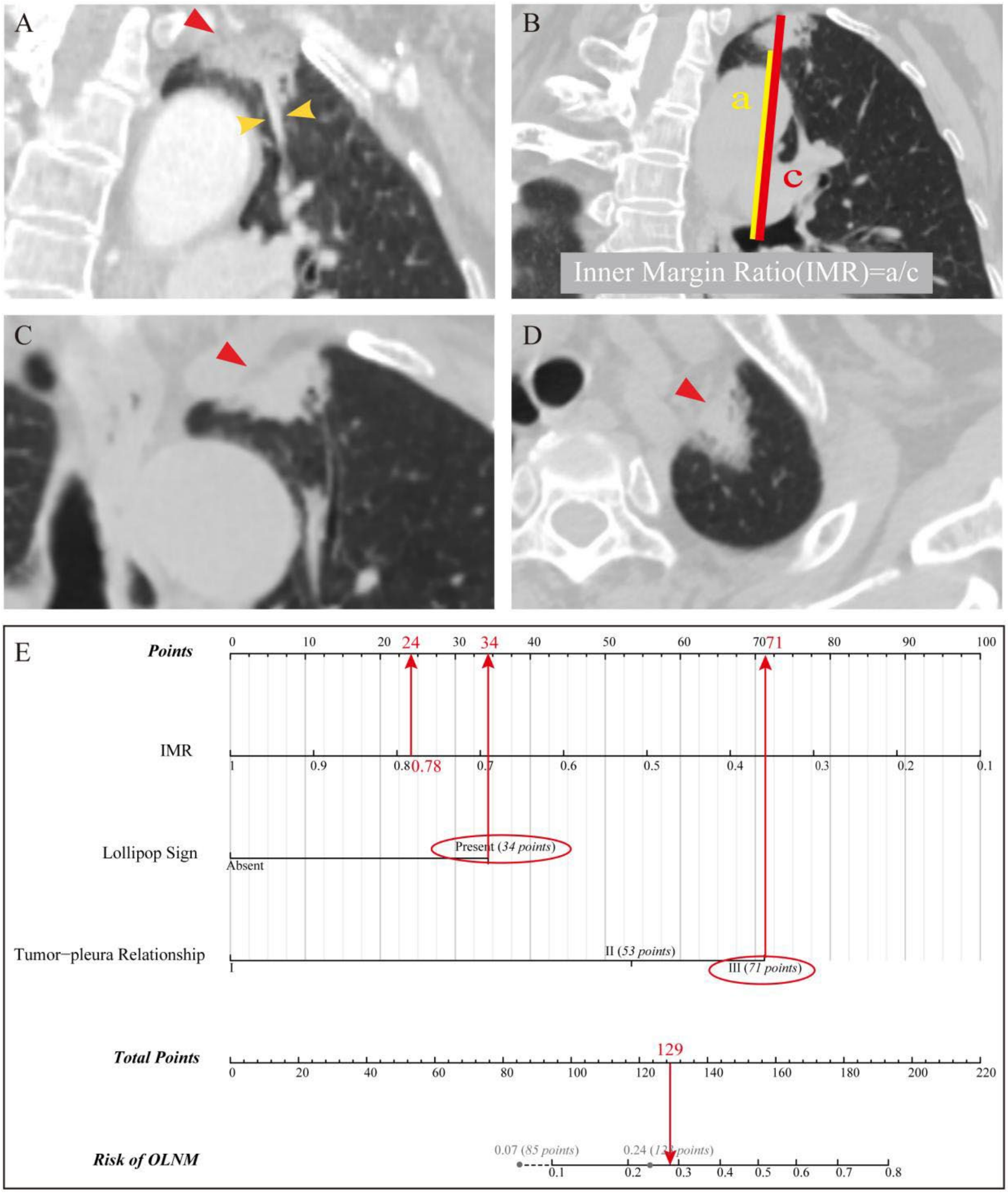

Figure S3: The key image features in 74-year-old female patient are shown in **(A-D)** CT images, who was confirmed by postoperative pathology as N2 lymph node metastasis. The application of nomogram based on inner margin ratio (IMR), lollipop sign, and tumor-pleura relationship is shown in **(E)**. When the model reached the highest area under the receiver operating characteristic curve of 0.81, the risk of occult lymph node metastasis (OLNM) was 0.24. When the model reached a sensitivity of 95%, the risk of OLNM was 0.07. **(A)** Lollipop sign, the red arrowhead indicates the lung nodule, and the yellow arrowheads indicate the single blood vessel entry. **(B)** IMR was 68.9mm/88.0mm = 0.78. **(C, D)** Tumor-pleura relationship type Ⅲ, the red arrowhead indicates the lung nodule adhered to the pleura. **(E)** Total points of nomogram was 129 points. Risk of OLNM was 0.28.

Supplemental materials undergo peer review but are not copyedited, and may include typographical errors.

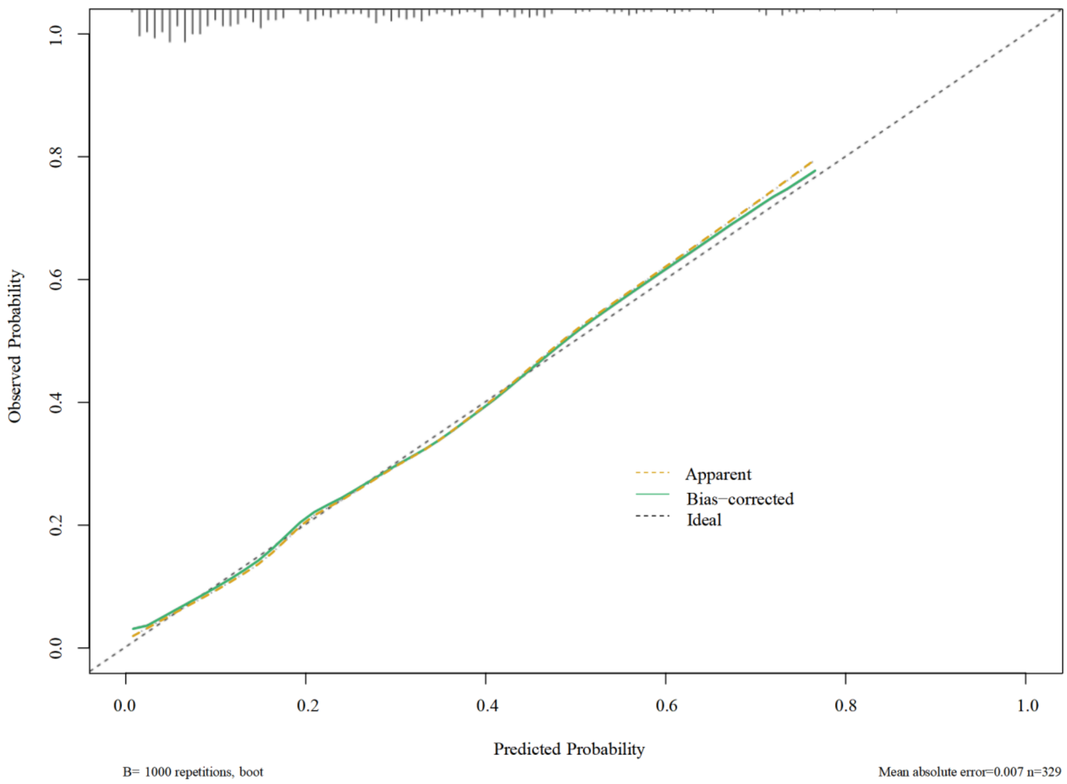

Figure S4: Calibration curve of the predictive model. The accuracy and calibration of the model were evaluated through internal validation using the bootstrap resampling method with 1000 repetitions (B = 1000). The bias-corrected (light green solid line) curve demonstrates a high degree of alignment with the ideal (black dashed line). The close agreement between the corrected predictions and the ideal line, along with a mean absolute error of 0.007 (n = 329), indicates that the model is well-calibrated, with its predicted probabilities accurately reflecting the observed outcomes.

Supplemental materials undergo peer review but are not copyedited, and may include typographical errors.

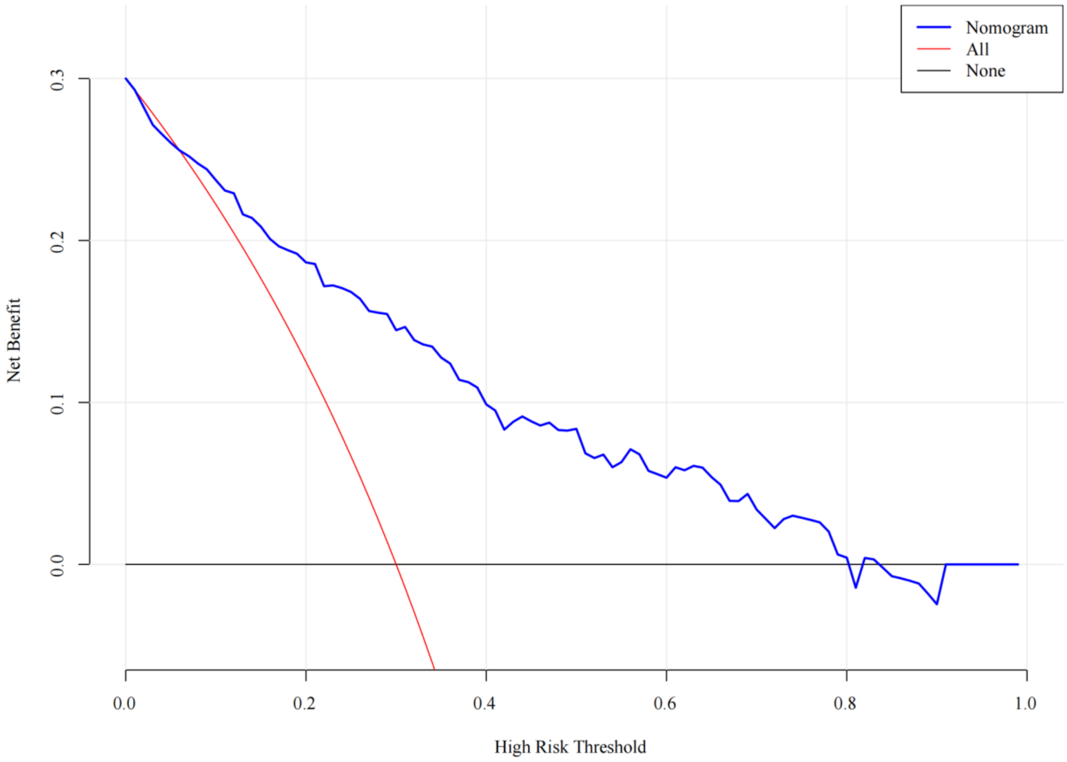

Figure S5: The decision curve analysis of the nomogram graphically shows the clinical practicability based on the threshold (*x*-axis) of predicting occult lymph node metastasis and the net benefit (*y*-axis) of using the model to stratify patients.

Supplemental materials undergo peer review but are not copyedited, and may include typographical errors.

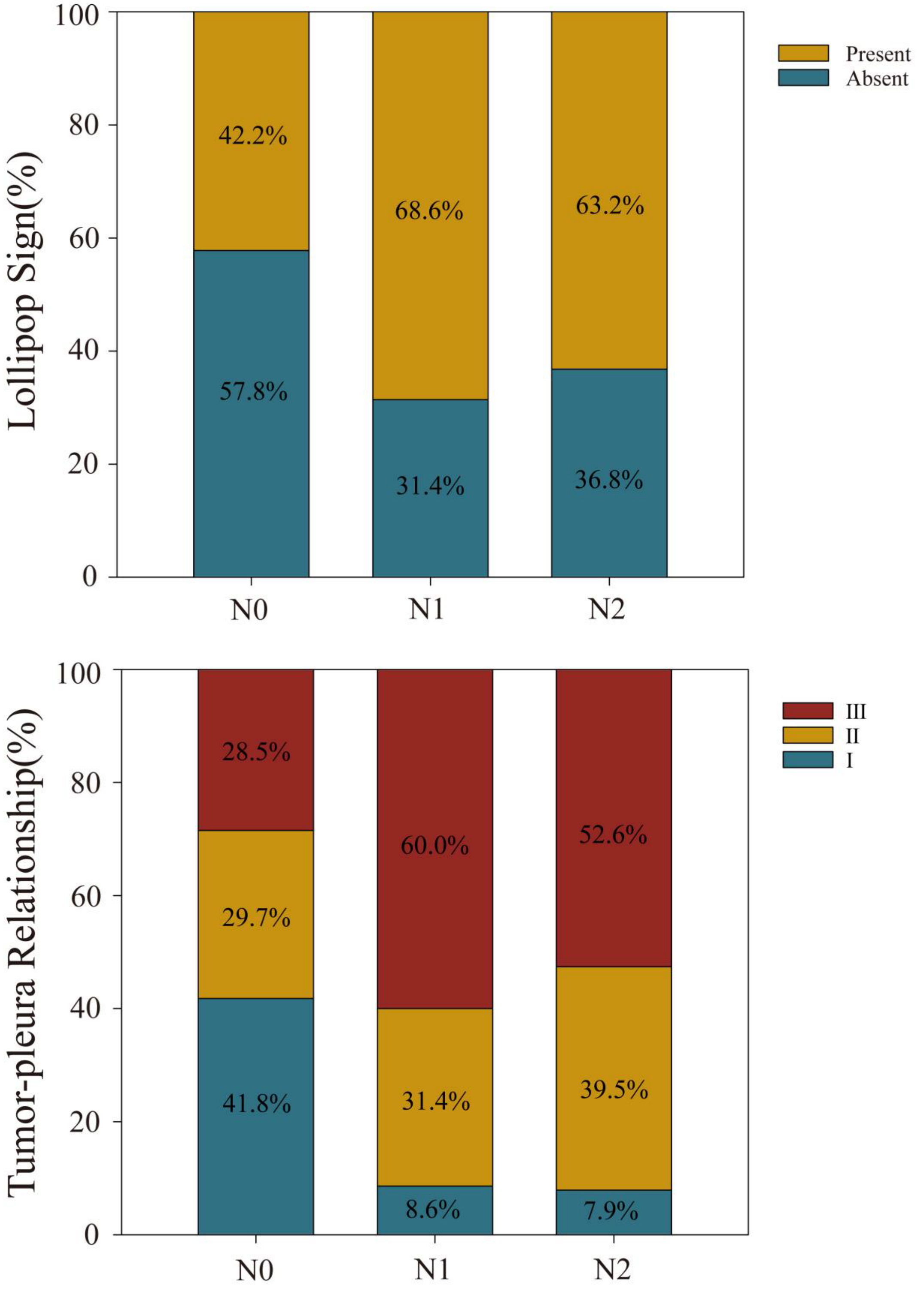

Figure S6: A percentage stacking chart of lollipop sign **(A)** and tumor-pleura relationship **(B)** based on three subgroups of N0, N1, and N2.

Supplemental materials undergo peer review but are not copyedited, and may include typographical errors.

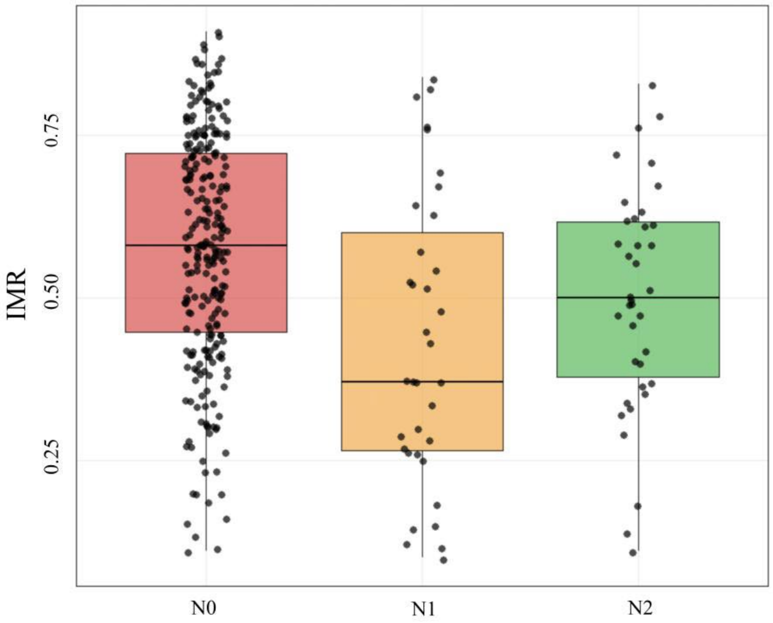

Figure S7: A box diagram of inner margin ratio (IMR) based on three subgroups of N0, N1 and N2.
